# Phytochemical Characterization of Bilberries and Their Potential as a Functional Ingredient to Mitigate Ochratoxin A Toxicity in Cereal-Based Products

**DOI:** 10.3390/nu16183137

**Published:** 2024-09-17

**Authors:** Denisia Pașca, Massimo Frangiamone, Luciano Mangiapelo, Pilar Vila-Donat, Oana Mîrza, Ana-Maria Vlase, Doina Miere, Lorena Filip, Jordi Mañes, Felicia Loghin, Lara Manyes

**Affiliations:** 1 Biotech AgriFood Lab, Faculty of Pharmacy and Food Sciences, University of Valencia, Burjassot, 46100 València, Spain; denisiai@alumni.uv.es (D.P.); massimo2.frangiamone@ext.uv.es (M.F.); jordi.manes@uv.es (J.M.); lara.manyes@uv.es (L.M.); 2 Department of Bromatology, Hygiene, Nutrition, Faculty of Pharmacy, “Iuliu Haţieganu” University of Medicine and Pharmacy, 6 Louis Pasteur, 400349 Cluj-Napoca, Romania; oana.stanciu@umfcluj.ro (O.M.); dmiere@umfcluj.ro (D.M.); lfilip@umfcluj.ro (L.F.); 3 Department of Toxicology, Faculty of Pharmacy, “Iuliu Haţieganu” University of Medicine and Pharmacy, 6 Louis Pasteur, 400349 Cluj-Napoca, Romania; floghin@umfcluj.ro; 4 Department of Pharmaceutical Sciences, University of Perugia, 06123 Perugia, Italy; luciano.mangiapelo@dottorandi.unipg.it; 5 Department of Pharmaceutical Botany, “Iuliu Hațieganu” University of Medicine and Pharmacy, 8 Victor Babes Street, 400347 Cluj-Napoca, Romania; gheldiu.ana@umfcluj.ro; 6 Academy of Romanian Scientists (AOSR), 3 Ilfov St, 050044 Bucharest, Romania

**Keywords:** mycotoxins, berries, in vitro digestion, mitigation, flow cytometry

## Abstract

Mycotoxin contamination of cereals and cereal-based products is a serious problem for food safety. Antioxidant-rich ingredients such as bilberries (*Vaccinium myrtillus* L., VM) may mitigate their harmful effects. Firstly, total phenolic content, antioxidant activity, and analytical phytochemical composition (hydroxycinnamic and hydroxybenzoic acids, flavanols, flavonols, and anthocyanins) were assessed in lyophilized wild bilberries from Romania. Secondly, this study evaluated bilberries’ effects on reducing ochratoxin A (OTA) bioaccessibility and cytotoxicity. An in vitro digestion model was developed and applied to four different types of bread: Control, VM (2%), OTA (15.89 ± 0.13 mg/kg), and OTA (16.79 ± 0.55 mg/kg)-VM (2%). The results indicated that VM decreased OTA bioaccessibility by 15% at the intestinal level. OTA-VM digests showed improved Caco-2 cell viability in comparison to OTA digests across different exposure times. Regarding the alterations in Jurkat cell line cell cycle phases and apoptosis/necrosis, significant increases in cell death were observed using OTA digests (11%), while VM addition demonstrated a protective effect (1%). Reactive oxygen species (ROS) analysis confirmed these findings, with OTA-VM digests showing significantly lower ROS levels compared to OTA digests, resulting in a 3.7-fold decrease. Thus, bilberries exhibit high potential as a functional ingredient, demonstrating protection in OTA mitigation effects.

## 1. Introduction

In recent years, mycotoxins have been thought to be a significant risk factor that can impact both human and animal health, posing one of the greatest risks to food and feed contamination. The term mycotoxins defines low-molecular-weight hazardous chemicals (0.3–0.7 kDa) generated by filamentous fungi, mainly *Fusarium* spp., *Penicillium* spp., and *Aspergillus* spp., and secreted as secondary metabolites. Mycotoxins occur naturally under pre- and post-harvest conditions and can contaminate dietary staple foods, particularly cereals throughout the production chain [1,2].

**Figure 1:**
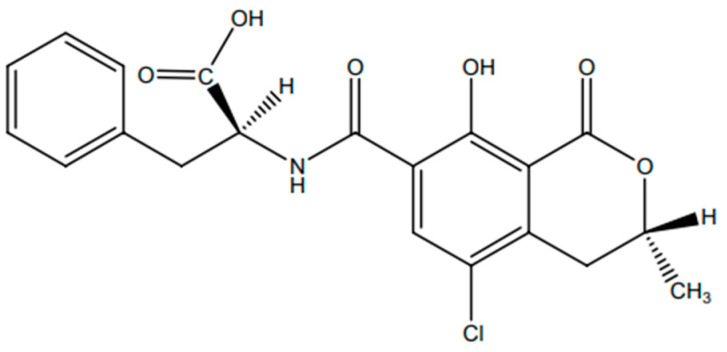
Chemical structure of OTA.

Ochratoxin A (OTA) is an odorless, thermostable, crystalline solid chemical agent with low water solubility and a molecular weight equal to 403.8 g/mol (Figure 1), predominantly produced by multiple *Aspergillus* and *Penicillum* species. In terms of biosynthesis, OTA is a pentaketide linked to β-phenylalanine that comes from the dihydrocoumarins family [3]. OTA has been detected in a wide range of products, such as cereals (wheat, barley, and rye) and their derivatives, as well as coffee, cocoa beans, chocolate, dried fruits, nuts, chili sauce, spices, various meats, dairy products including milk and cheese, and wine and beer, all of which are common dietary staples worldwide [4]. OTA is known for its negative effects on a range of in vivo and in vitro models, which include immunosuppressive, genotoxic, neurotoxic, teratogenic, and embryotoxic effects [5]. The high contamination rate of OTA and its thermal stability make it very difficult to remove it from the food chain. The presence of OTA is crucial in warm and humid climates, where toxigenic filamentous fungi can flourish. Due to its ubiquitous presence across diverse sources and its potential for bioaccumulation within the chain of food, OTA poses a significant risk to both human and animal health [6]. Regulatory limits have been established to reduce OTA in food products. Thus, European regulatory agencies and organizations have set maximum limits for OTA levels in unprocessed cereals (5.0 μg/kg), cereal-based products (3.0 μg/kg), cocoa powder (3.0 μg/kg), roasted coffee (3.0 μg/kg), dried fruit, except raisins and figs (2.0 μg/kg), and wine (2.0 μg/kg) [7].

Reducing the harmful impact on human and animal health and on the environment is known as mitigation [8,9]. One of the strategies to reduce the toxic effects produced by mycotoxin ingestion is to increase functional ingredients in human and animal diets. Berries are widely consumed fruits that are rich in both nutritive and non-nutritive components, such as vitamins, minerals, and polyphenols [10]. Berries, and specifically their phenolic compounds, include anthocyanins, which exhibit a range of health benefits, including anti-inflammatory, antioxidant, anti-diabetic, anti-cancer, cardio-protective, neuroprotective, antimicrobial, and anti-obesity properties [11]. In the case of bilberry (*Vaccinium myrtillus* L. species), the polyphenol-rich extract of the fruits demonstrated protective action against oxidative DNA damage and inhibitory actions against lipid peroxidation. When compared to the cultivated genotypes of blueberries, bilberries showed a two- to three-fold higher antioxidant capacity and a higher concentration of bioactive components that substantially improved health [12]. Research using both cell and animal models has demonstrated that bilberry has anti-inflammatory properties. It also reduces the expression of IL-6, IL-1β, and tumor necrosis factor-α, inducing nitric oxide synthases and cyclooxygenases, and altering the nuclear factor kappa B and Janus kinase-signal transducer and activator of transcription signaling pathways [13]. Numerous research studies have analyzed the protective impact of antioxidant compounds against OTA, but few of them have investigated the effect of natural compounds from specific functional ingredients [14,15].

The bioaccessibility of mycotoxins is a crucial component in the risk assessment process for human health. This refers to the proportion of a nutrient, bioactive compound, or toxin that enters the gastrointestinal tract prepared for absorption after being liberated from the food matrix [16]. In similar studies that have carried out in vitro human digestion models, a significant decrease in mycotoxin bioaccessibility was seen when compounds with antifungal and antimicrobial properties were incorporated [15]. One of the main interests of this research was to investigate the effects of these compounds at the intestinal level. Mycotoxins have been demonstrated by other authors to disrupt the intestinal physical barrier. In order to most accurately represent the intestinal physical and chemical barrier in vitro, the Caco-2 cell line was considered to be the most adequately represented, as it spontaneously differentiates to form polarized monolayers after seeding for 21 days [17].

OTA exposure has been linked to gastrointestinal barrier impairment, primarily replicated with Caco-2 cells, as evidenced by numerous in vitro studies. Cell cycle modifications, apoptosis, and the production of intracellular reactive oxygen species (ROS) are the causes of this impairment [18]. Additionally, OTA can penetrate the bloodstream and change the permeability of the gut barrier, which may lead to significant changes in immune cell homeostasis. One appropriate immunological model for studying human conditions in vitro is the Jurkat T cell line. These cells exhibit significant OTA cytotoxicity, which has been related to metabolic reprogramming and glycolysis activation [19].

Thus, this study aimed to assess the effects of bilberries on reducing the bioaccessibility and cytotoxicity of OTA, as well as the possible protective effect of the chosen natural ingredient in vitro, using intestinal digests obtained by a simulated digestion model. The innovation of this work consists of studying the effects of OTA when an antioxidant-rich ingredient, bilberries (*Vaccinium myrtillus* L.), is incorporated as a nutritional component in food products.

## 2. Material and Methods

### 2.1. Chemicals and Reagents

Sodium carbonate, potassium chloride, Trolox (6-hydroxy-2,5,7,8-tetramethylchroman-2-carboxylic acid, 97%), DPPH (2,2-diphenyl-1-(2,4,6-trinitrophenyl) hydrazine), and dimethyl sulfoxide (DMSO) were purchased from Sigma Aldrich (Schnelldorf, Germany). Folin–Ciocâlteu reagent, gallic acid, and methanol (MeOH) were obtained from Merck (Darmstadt, Germany). All standards for spectrophotometric assays and Liquid Chromatography–Mass Spectrometry (LC–MS) analysis were sourced from Sigma Aldrich (Schnelldorf, Germany). All reagents were of analytical grade, and High-Performance Liquid Chromatography (HPLC)-grade solvents were utilized.

Strength wheat flour (Alteza) was obtained from Haricaman, S.L. Ctra (Toledo, Spain), barley grain (Biogra) was obtained from P.D.R. Sorribas S.A.U., Polinya (Valencia, Spain), fresh yeast (Levanova) was produced by Lesaffre Iberica S.A. (Valladolid, Spain), sea salt was produced by Polasal S.A. (Alicante, Spain), and white sugar was obtained from Pfeifer & Langen GmbH & Co (Colonia, Germany).

MeOH and acetonitrile (ACN) were supplied by Fisher Scientific (Madrid, Spain). An OTA standard solution (purity > 99%) was obtained from Sigma-Aldrich (St. Louis, MO, USA). A stock solution was prepared in MeOH at a concentration of 100 µg/mL, followed by serial dilutions. All working solutions were stored at −20 °C and protected from light.

Potassium chloride (KCl), potassium thiocyanate (KSCN), sodium bicarbonate (NaHCO3), sodium hydroxide (NaOH), hydrochloric acid (HCl), MTT (3-(4,5-dimethylthiazol)-2,5-diphenyltetrazolium), sodium dihydrogen phosphate (NaH_2_PO_4_), sodium chloride (NaCl), sodium sulfate (Na_2_SO_4_), urea (CO(NH_2_)_2_), α-amylase (930 U/mg A3403), pepsin A (674 U/mg P7000), pancreatin (762 U/mg P1750), the ROS detection kit (H_2_-DCFDA), tert-butyl hydroperoxide (TBHP) solution, MitoParaquat, and bile salts (B8631) were sourced from Sigma-Aldrich (St. Louis, MO, USA). Deionized water was supplied by a Milli-Q water purification system (Millipore, Bedford, MA, USA). Dimethyl sulfoxide (DMSO), the MitoTracker Green M7514 kit (Invitrogen), the MitoSOX kit for mitochondrial ROS generation, and the Caco-2 culture medium DMEM were acquired from Thermo Fisher Scientific (Waltham, MA, USA). Phosphate-buffered saline (PBS) was sourced from Sigma Chemical Co. (St. Louis, MO, USA). The Jurkat culture medium RPMI was obtained from Biowest (Nuaillé, France), while the Annexin V-FITC kit was purchased from Miltenyi Biotec (Bergish Gladbach, Germany). The CycleTEST™ PLUS DNA Reagent Kit and propidium iodide (PI) were supplied by BD Biosciences (San Diego, CA, USA).

### 2.2. Functional Ingredient

Bilberries were harvested from the spontaneous flora of a Romanian mountainous area (Maramureș Mountains, Romania) in September 2022 and were identified by Dr. Ramona Paltinean from the Department of Pharmaceutical Botany, Iuliu Hațieganu University of Medicine and Pharmacy as a *Vaccinium myrtillus* L. species, voucher specimens being kept in the herbarium of the Department (voucher no. 106.7.2.1). Bilberries were immediately frozen at −20 °C and stored until the experiment was conducted, as freezing is considered the most appropriate method for preserving the bioactive compounds of the functional ingredient [20]. In order to achieve a homogeneous powder, bilberries were lyophilized using the Advantage 2.0 apparatus from SP Scientific (Warminster, PA, USA) for 24 h at −55 °C, and afterward for 48 h at −25 °C under 200 mTorr of pressure. The ingredient was ground using an Electric Grinder (Network One Distribution SRL) and stored as a lyophilized powder at room temperature until their use (about two weeks), according to the literature [21]. For the lyophilized matrix, the total phenolic content (TPC) and total antioxidant activity (TAA) were evaluated using the DPPH assay, and LC–MS analysis was performed.

#### 2.2.1. Extract Preparation

To prepare the extract, we used an experimental method designed by Marzullo et al. [22]. Thus, 3 g of lyophilized bilberry powder, in three replicates, was extracted using MeOH/H_2_O solvent (56:44 *v*/*v*). Ultrasound-assisted extraction (UAE) was performed for 17 min, followed by centrifugation at 3000 rpm for 15 min. The supernatant was filtered and used in the performed experiments.

#### 2.2.2. Total Phenolic Content (TPC)

The total phenolic content (TPC) in the extract was determined using a spectrophotometric method with Folin–Ciocâlteu (FC) reagent, following the protocol of Vlase et al. [23]. Specifically, a 20 µL sample aliquot was placed in a 96-well plate and combined with 80 µL of FC reagent, diluted at a 1:10 ratio. After a 3-min incubation, 80 µL of a 7.5% (*w*/*v*) sodium carbonate solution was added. Absorbance was measured at 760 nm against a blank reagent after a 30-min incubation in the dark at room temperature using a Synergy HT microplate reader (BioTek Instruments, Inc., Winooski, VT, USA). A calibration curve was created using gallic acid (GA) as the reference standard (0.01–0.16 mg/mL, r^2^ = 0.9986), and the results were expressed as mg of gallic acid equivalents (GAE) per gram of dry weight (DW).

#### 2.2.3. Determination of Antioxidant Activity

The DPPH free radical scavenging activity was evaluated using a 96-well plate by mixing 10 µL of the extract with 9 µL of a 0.004% methanolic DPPH solution, followed by a 30-min incubation in the dark. The change in absorbance was measured at 517 nm, and the DPPH reduction was calculated in relation to the Trolox stock solution using the formula A = A_stock solution_ − A_sample_, where A_stock solution_ represents the absorbance of the DPPH radical with methanol, and A_sample_ represents the absorbance of the DPPH radical mixed with the plant extract. Absorbance values were compared to a solvent blank using a microplate reader. Trolox reagent was used as the reference standard (0.025–0.3 mg/mL, r^2^ = 0.9942), and results were expressed as mg Trolox equivalents (TE) per gram of dry weight (DW).

#### 2.2.4. Phytochemical Analysis by LC–MS

The phytochemical composition of the VM extract was examined using HPLC–MS with a validated analytical method [24]. Quantification of the target compounds was conducted using an Agilent 1100 HPLC Series system (Agilent, Santa Clara, CA, USA), which included a degasser, binary gradient pump, column thermostat, autosampler, and UV detector. The HPLC system was coupled with an Agilent 1100 mass spectrometer detector (HPLC/MSD Ion Trap VL). A reverse-phase analytical column (Zorbax SB-C18, 100 × 3.0 mm i.d., 3.5 μm particle) was used for separation at a working temperature of 48 °C. Compound detection was achieved in both UV and MS modes, with the UV detector set at 330 nm until 17.5 min, and then at 370 nm. The MS system used an electrospray ion source in negative mode. Chromatographic data were analyzed with ChemStation (vB01.03) and DataAnalysis (v5.3) software from Agilent, Santa Clara, CA, USA.

### 2.3. Flour Contamination and OTA Production

Barley flour was naturally contaminated by the fungal species A. steynii 20510, obtained from the Spanish Collection of Type Crops (CECT) at the Science Park of the University of Valencia (Paterna, Spain). For this, 400–450 g of barley was placed into 1 L glass jars that had been previously autoclaved. The cereals were then inoculated with 15–20 mL of a spore and mycelium suspension in peptone water containing 0.1% Tween 80 (Thermo Fisher Scientific, Waltham, MA, USA), prepared from the corresponding fungal strain. The jars were kept at room temperature in the dark for one month. Following the incubation period, the cereals were autoclaved to eliminate fungal contamination and then ground into flour to ensure complete homogenization. Mycotoxin levels in the contaminated flour were measured by Liquid Chromatography coupled with a Fluorescence Detector (LC–FLD) after solid–liquid extraction, as detailed in Section 2.3.2.

#### 2.3.1. OTA Extraction and Bread Analysis

To determine the OTA concentration, an extraction was performed using a MeOH/H_2_O solvent mixture (80:20 *v*/*v*). For this, 10 g of bread or 5 g of contaminated barley flour was ground using an Ultraturrax (T 18 digital ULTRA-TURRAX^®^, Staufen, Germany) for 3 min, followed by centrifugation (Centrifuge 5810R, Eppendorff, Hamburg, Germany) at 4500 rpm for 5 min.

The LC-FLD analysis was conducted under the conditions described in Section 2.3.2. The supernatant was collected and filtered using a 0.22 μm syringe filter (Phenomenex, Madrid, Spain), and each sample was injected into screw-thread vials in triplicate. Matrix-matched calibration curves were created by spiking OTA standard solution at various concentrations into non-contaminated barley flour or C-bread extract.

#### 2.3.2. LC-FLD Quantitative Analysis

In order to perform the LC-FLD quantitative analysis of OTA, the protocol described by Vila-Donat et al. [25] was carried out. To quantify OTA concentration, an Agilent 1100 quaternary pump series (Agilent Technologies, Santa Clara, CA, USA) was used, connected to an Agilent 1200 FLD (Agilent Technologies), which included a vacuum degasser and an automatic sampler (Agilent Technologies). The stationary phase consisted of a Kinetex EVO C18 column (150 × 4.6 mm, 5 µm particle size, 100 Å pore size by Phenomenex, Palo Alto, CA, USA). An isocratic elution method was employed, using ACN/H_2_O/CH_3_COOH (55/43/2 *v*/*v*) as the mobile phase, with a flow rate of 0.8 mL/min. The column was preconditioned with the mobile phase for 20 min prior to use and maintained at 40 °C during the analysis. Injection volumes were 20 µL for flour and bread extracts and 40 µL for gastric and intestinal digests. High linearity, reflected by r^2^ values greater than 0.991, was achieved for all matrix-matched calibration curves. The excitation and emission wavelengths for OTA were set at 330 nm and 460 nm, respectively. The reliability of the LC-FLD method was confirmed through the determination of the limit of detection (LOD) and limit of quantification (LOQ) (Table 1).

### 2.4. Bread Preparation and Baking

Breads were produced using the ingredients and methodology explained by Mangiapelo et al. [26]. Bilberries as an ingredient were combined with and without contaminated barley flour to prepare four breads: wheat flour bread (named Control (C)), bread with wheat flour and *Vaccinium myrtillus* L. 2% (named VM), bread with wheat flour contaminated with OTA (named OTA), and bread with wheat flour contaminated with OTA and *Vaccinium myrtillus* L. 2% (named OTA-VM). The breads were prepared by mixing the ingredients listed in Table 2.

### 2.5. In Vitro Static Digestion Model

An in vitro digestion model was used to simulate the entire human digestion process in stages for the studied samples, following a protocol previously described by Lázaro et al. [27]. This process included three digestion phases: oral, gastric, and intestinal. Sterile Stomacher^®^ bags (IUL, Barcelona, Spain) were filled with 10 g of ground bread, 6 mL of artificial saliva, and 84 mL of 37 °C Milli-Q water, and then mixed for 30 sec using an IUL Stomacher (IUL S.A, Barcelona, Spain) to mimic the mastication process. The artificial saliva was prepared by combining 1 mL each of KSCN (20 g/L), KCl (89.6 g/L), Na_2_SO_4_ (57 g/L), NaH_2_PO_4_ (8.8 g/L), 0.17 mL of NaCl (175.3 g/L), 2 mL of NaHCO_3_ (84.7 g/L), 0.8 mL of urea (20 g/L), 2.5 mg of mucin, and 29 mg of α-amylase, followed by dilution with distilled water to a final volume of 100 mL. The saliva pH was adjusted to 6.8 ± 0.2. After simulating the oral phase, the mixture was transferred to an Erlenmeyer flask for the gastric phase. The pH was reduced to 2 using 6 N HCl, and a pepsin solution (1 g of pepsin in 25 mL of 0.1 N HCl) was added. The mixture was then incubated in the dark with continuous stirring at 100 rpm for 2 h at 37 °C using an orbital shaker (Infors AG CH-4103, Bottmingen, Switzerland). After incubation, an aliquot of the gastric digest was stored at −20 °C for further analysis. To replicate intestinal digestion, the pH was adjusted to 6.5 ± 0.2, followed by the addition of bile salts and a pancreatic solution (0.1 g of pancreatin and 0.625 g of bile salts in 25 mL of 0.1 N NaHCO_3_). The digests were incubated as formerly detailed (2 h at 37 °C in darkness and slight agitation), the samples were centrifuged at 3000 rpm for 5 min (Centrifuge 5810R, Eppendorf, Germany), and the supernatant containing the intestinal digests was collected and stored in darkness at −20 °C. The entire process was repeated three times for each bread variety (*n* = 3).

### 2.6. Gastrointestinal Extracts Analysis and Bioaccessibility

The gastric and intestinal digests were filtered through a 0.22 µm filter and then analyzed using LC–FLD for OTA determination, as detailed in Section 2.3.2. Standard calibration curves were created in MeOH (1–1000 µg/L) using the OTA standard (1000 mg/L). For quantification, matrix-matched calibration curves were prepared for each bread condition (C, VM, OTA, OTA-VM) by spiking blank digested extracts with OTA at equivalent concentrations. OTA bioaccessibility (%) was determined as the percentage of mycotoxins from the initially digested bread that was detected in the digested extracts. The amount of mycotoxin (µg) in 10 g of bread (A) was calculated from the bread concentration (µg/kg) using conversion factors (×10/1000). The amount of mycotoxin (µg) in 100 mL of digest (B) was calculated from the digest concentration (µg/L) using conversion factors (×100/1000). To find the bioaccessibility percentage, these quantities were related as B/A×100. The bioaccessibility was then directly calculated using the formula provided in Equation (1):

Bioaccessibility = digest concentration (µg/L) * 1000/bread concentration (µg/kg)(1)

### 2.7. Cell Cultures

Human colon carcinoma Caco-2 cells (ATCC HB-8065) were grown in a monolayer in 75 cm^2^ polystyrene tissue culture flasks at a density of 25,000 cells/cm^2^. The complete growth medium used was DMEM with 4.5% glucose, supplemented with 10% fetal bovine serum (FBS), 1% HEPES, 1% non-essential amino acids (NEAA), 1% penicillin/streptomycin, and 0.1% fungizone. The cells were incubated under conditions of pH 7.4, 37 °C, 5% CO_2_, and 95% humidity, maintained using a Thermo Forma SteriCycle CO_2_ incubator (Thermo Scientific, MA, USA). The cell media was refreshed every 2–3 days.

Jurkat cells (ATCC-TIB152), originating from human T lymphocytes in peripheral blood, were cultured in RPMI-glutamax medium supplemented with 10% (*v*/*v*) inactivated fetal bovine serum (FBS), 100 U/mL penicillin, and 100 mg/mL streptomycin. The cells were incubated at pH 7.4, 37 °C with 5% CO_2_ and 95% air atmosphere at constant humidity. The culture medium was replaced every 2–3 days.

### 2.8. Cell Viability Assay

The viability of differentiated Caco-2 cells was assessed using the MTT assay following exposure to contaminated bread extracts. This assay was conducted based on the protocol outlined by Manyes et al. [18], with minor adjustments. For this study, Caco-2 cells were cultured in 24-well tissue culture plates, with 500 μL of a 1 × 10^5^ cells/mL suspension added to each well. The medium was changed every 2–3 days until day 21, the period necessary for Caco-2 cells to establish the in vitro gastrointestinal barrier. Five exposure times (24 h; 48 h; 72 h; 96 h; and 120 h) were considered, using different dilutions of intestinal digests (not diluted; 1/2; 1/4; 1/8; 1/16; and 1/32 *v*/*v*). For the experiments involving cell cultures, the concentration of OTA was standardized for both the OTA digest and the OTA-VM digest at equivalent concentrations. After each exposure time, each well received 250 μL MTT solution (1 mg/mL of MTT solution), followed by 4 h of incubation (37 °C in darkness). Afterward, MTT was discarded, and 400 μL of DMSO was added to each well. Absorbance was measured at 620 nm using the Multiskan EX Enzyme-Linked Immunosorbent Assay (ELISA) plate reader (Thermo Scientific, MA, USA). Cell viability was expressed as a percentage (%) relative to the control cells.

### 2.9. Flow Cytometry Analysis

All flow cytometry assays were conducted using a MACSQuant 16 analyzer (Miltenyi Biotech GmbH, Bergisch Gladbach, Germany). The lasers used were blue (488 nm), violet (405 nm), and red (640 nm). Using a 525/50 FITC B1 filter, the fluorescence results of Annexin V, MitoTracker, and H_2_-DCFDA were collected. Cell cycle fluorescence data were obtained using a 579/34 PE (B2) filter and mitoSOX fluorescence data were obtained using a V4 channel with a 615/20 filter. A minimum of 20,000 events for each one were taken into consideration.

#### 2.9.1. Cell Cycle Analysis

The cell cycle profile was analyzed by staining the DNA with a fluorescent dye and measuring its intensity. Jurkat cell nuclei were stained using a BD Cycletest™ Plus DNA Reagent kit (BD Biosciences, Franklin Lakes, NJ, USA). Flow cytometry was employed to determine the distribution and estimate the percentage of cells in various phases of the cell cycle. After exposing Jurkat cells to intestinal digests for 7 days, they were collected and centrifuged. The cell pellet was resuspended in a buffer solution. For staining, the cells were first incubated with solution A (trypsin buffer) for 10 min at room temperature, followed by a second incubation with solution B (trypsin inhibitor and RNase buffer). Staining was performed with PI stain solution for 10 min in the refrigerator (2–8 °C) in the dark. The samples were analyzed via flow cytometry with the appropriate settings.

#### 2.9.2. Apoptosis/Necrosis Analysis

To measure the percentage of cells undergoing apoptosis or necrosis, an annexin V-FITC Kit (BD Biosciences, USA) was utilized. After exposing Jurkat cells to diluted intestinal digests (1/10) for 7 days, the cells were collected in a centrifuge tube and washed with binding buffer. The samples were then centrifuged at 300 G for 10 min, and the cell pellet was resuspended in a binding buffer with 5 μL of annexin V-FITC. The cells were incubated for 15 min at room temperature in the dark. Finally, the cells were washed with binding buffer and stained with PI solution before analysis by flow cytometry.

#### 2.9.3. ROS Analysis

After 7 days of exposure to intestinal digests, the Jurkat cells were collected into a centrifuge tube and centrifuged for 5 min at 300 G. Then, 5 μM H_2_-DCFDA solution prepared in a fresh medium was added to resuspend the cell pellet, which was then incubated for 20 min at 37 °C in the dark. The samples were subsequently washed twice with PBS and analyzed using a flow cytometer. As a positive control, 1 mM of tert-butyl hydroperoxide (TBHP) was used for 30 min of incubation.

#### 2.9.4. Mitochondrial Mass and Mitochondrial ROS Analysis

MitoTracker green dye was used to assess mitochondrial mass. Following 7 days of exposure to intestinal digests, 1–3 × 10^5^ Jurkat cells were collected and centrifuged at 300 G for 5 min. After being resuspended in a staining solution containing a 100 nM MitoTracker probe, the cell pellet was incubated at 37 °C for 20 min in darkness. Samples were centrifuged, resuspended in PBS, and then examined using flow cytometry. MitoSOX reagent (1 μM as the final concentration) was used to track the formation of ROS in the mitochondria of Jurkat cells. Using a 15 mL centrifuge tube, the 1–3 × 10^5^ Jurkat cells were collected and centrifugated. To every sample, mitoSOX reagent was applied. Samples were examined using a flow cytometer after being washed in PBS. Additionally, 50 μM of MitoParaquat solution was utilized as a positive control and it was incubated for 16 h.

### 2.10. Statistical Analyses of the Data

The results from the independent experiments were reported as mean ± SD. Statistical comparisons of the results were performed using a paired Student’s *t*-test. The statistical software used was Microsoft Excel^®^ for Microsoft 365 MSO (Microsoft Office Professional Plus 2021). Statistics were judged significant at *p*-values less than 0.05. For the flow cytometry tests, MACSQuantify version 2.1 was used. The graphs were created using GraphPad Prism 6.01 for Windows (Boston, MA, USA) and Microsoft Excel (version LTSC 2021).

## 3. Results

### 3.1. Analysis of the Functional Ingredient

Table 3 summarizes the results obtained for the spectrophotometric assays performed on the lyophilized bilberry extract.

Table 4 shows the quantification results for the individual phenolic acids and flavonoids identified in the extracts of the bilberry species studied.

### 3.2. Analysis of Sample Breads and Contaminated Barley Flour

Table 5 shows the concentration of the mycotoxin obtained in contaminated flour, bread, gastric, and intestinal digests. No OTA was detected in C and VM breads.

### 3.3. OTA Bioaccessibility in Gastric and Intestinal Digests

**Figure 2:**
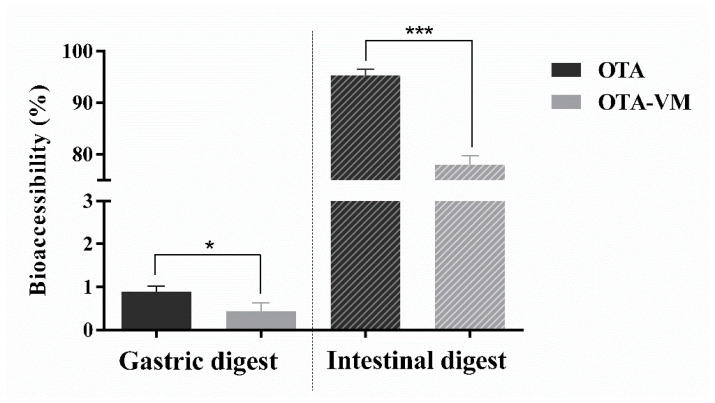
Gastric and intestinal OTA bioaccessibility (%) calculated after the in vitro simulated digestion (n = 3). Significant differences between OTA and OTA-VM are indicated as *p* < 0.05 (*), *p* < 0.001 (***). Bread with Ochratoxin A (OTA); bread with Ochratoxin A + *Vaccinium myrtillus* L. 2% (OTA-VM).

OTA bioaccessibility was considerably lower in gastric digests compared to intestinal digests, with a significative difference between OTA and OTA-VM gastric digests (*p*-values < 0.05) ranging from 0.27% to 0.66% of OTA bioaccessibility. In contrast, a significant difference (*p*-values < 0.001) was observed between OTA and OTA-VM in the intestinal digest, showing a reduction of 14–19% in OTA when the functional ingredient was included (Figure 2).

### 3.4. Cell Viability Results

**Figure 3:**
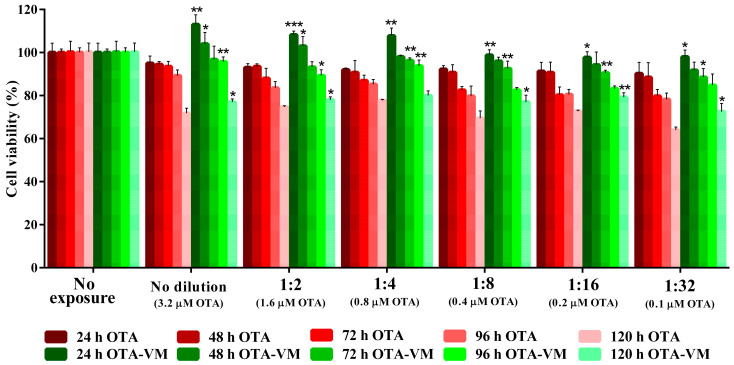
Cell viability in differentiated Caco-2 cells after exposure to various dilutions of intestinal digests (3.2 μM OTA for no dilution) over five different time points. The data are presented as mean ± SD (n = 4). Significant differences between OTA and OTA-VM intestinal digests at the same dilution and exposure time are denoted as *p* < 0.05 (*); *p* < 0.01 (**); *p* < 0.001 (***). OTA: bread with wheat flour and barley contaminated with Ochratoxin A; OTA-VM: bread with wheat flour and barley flour contaminated with Ochratoxin A and *Vaccinium myrtillus* L. 2%.

Figure 3 illustrates the toxic effects of OTA on Caco-2 cell viability. It was demonstrated that the addition of bilberries (OTA-VM) in contrast to the OTA digest contributed to significantly augmenting cell viability in every exposure time at some specific concentrations. As an average, for OTA exposure at 24 h, a decrease in viability of up to 10% was observed, while for OTA-VM at 24 h, viability decreased by 2%. The same trend was observed at 48 h exposure, with a significant of 10% increase when VM was added. After 72 h exposure, a reduction in cell viability of up to 20% was observed in digested OTA, while the most significant decrease in digested OTA-VM was observed at 1/32 dilution, with a percentage of 12%. The same trend was observed in the case of exposure to 96 h. With 120 h exposure, the most substantial reduction in viability was observed for OTA (up to a 36% decrease).

Comparing OTA and OTA-VM digests at every exposure time, a significant cell viability increase of 15–18% for the exposure to no diluted digest, 1/2 and 1/4 dilution digests for 24 h, of 3–10% for 48 h, of 5–10% for 72 h, of 3–9% for 96 h, of 2–9% for 120 h was observed when OTA-VM was used (Figure S1).

### 3.5. Cell Cycle Analysis

**Figure 4:**
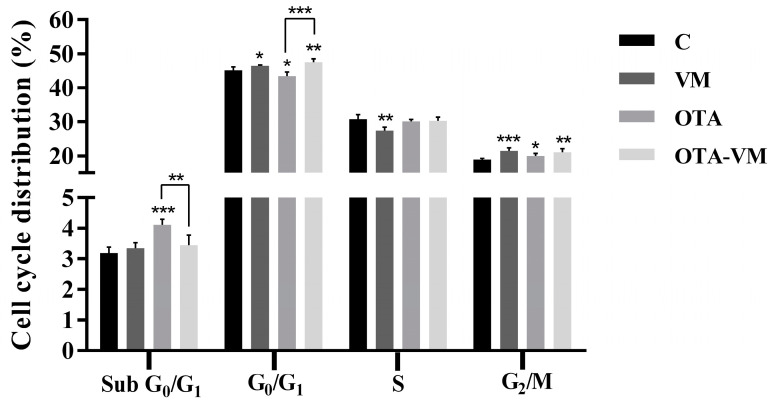
Effect of 1/10 intestinal bread digests (0.32 μM OTA) exposure for 7 days on Jurkat cells cycle phases (Sub G_0_/G_1_; G_0_/G_1_; S; G_2_/M). Data are presented as mean ± SD (n = 4) and significant differences between intestinal digests (VM, OTA, and OTA-VM) and control or OTA and OTA-VM are indicated as *p* < 0.05 (*); *p* < 0.01 (**); *p* < 0.001 (***). The acronyms for cell cycle phases are G for growth, S for DNA synthesis, and M for mitosis. C: wheat flour bread; VM: bread with wheat flour and *Vaccinium myrtillus* L. 2%; OTA: bread with wheat flour and barley flour contaminated with Ochratoxin A; OTA-VM: bread with wheat flour and barley flour contaminated with Ochratoxin A and *Vaccinium myrtillus* L. 2%.

A PI staining kit was employed to analyze the effect of intestinal digests on the cell cycle. Figure 4 shows that cells exposed to OTA digest exhibited a significant increase in arrest at the G_0_/G_1_ phase compared to the control and the OTA-VM digest. The same tendency was observed for the G_0_/G_1_ phase. In contrast, there was no difference in the distribution of cells in the S phase between cells exposed to OTA-VM digest and the control. In the G_2_/M phase, significant differences in cell distribution were observed between OTA and OTA-VM compared to the control. Differences in % were too small to be considered biologically significant.

### 3.6. Apoptosis/Necrosis Analysis

**Figure 5:**
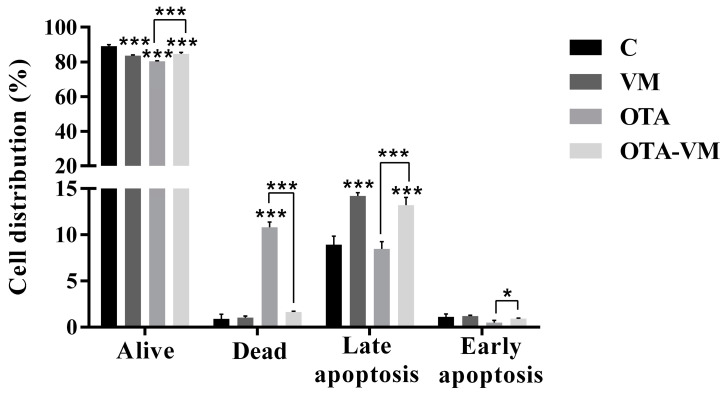
Effect of intestinal digests (0.32 μM OTA) exposure on Jurkat cells after 7 days on the apoptosis and necrosis pathway. Significant differences between intestinal digests (VM, OTA, and OTA-VM) and control or OTA and OTA-VM are indicated as *p* < 0.05 (*); *p* < 0.001 (***) and data graph bars represent the mean ± SD (n = 4). C: wheat flour bread; VM: bread with wheat flour and *Vaccinium myrtillus* L. 2%; OTA: bread with wheat flour and barley flour contaminated with Ochratoxin A; OTA-VM: bread with wheat flour and barley flour contaminated with Ochratoxin A and *Vaccinium myrtillus* L. 2%.

Figure 5 shows the effect of 1/10 intestinal digests on cell apoptosis/necrosis. The results demonstrated a significant difference in alive cells for all the digests (83.68 ± 0.43% for VM, 80.46 ± 0.28% for OTA, and 84.63 ± 0.82% for OTA-VM) compared to the control (89.05 ± 0.89%). For dead cells, a significant difference was identified in the case of exposure with OTA intestinal digest (10.79 ± 0.58%) compared with the control (0.91 ± 0.49%) and compared with the OTA-VM exposure (1.63 ± 0.09%). For the cells undergoing late apoptosis, significant differences were shown for VM (14.20 ± 0.33%) and OTA-VM (13.17 ± 0.85%) intestinal digests, and also in OTA compared with OTA-VM exposure. A minimal distribution of cells in early apoptosis was observed across all samples (Figure S2).

### 3.7. ROS and Mitochondrial ROS Analysis

**Figure 6:**
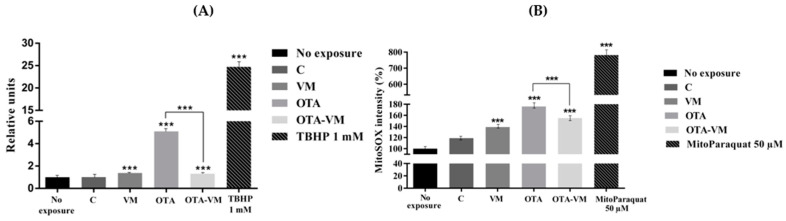
Reactive oxygen species (ROS) measured by flow cytometry using Jurkat cells after 7 days of exposure. (**A**) Effect of intestinal digests (0.32 μM OTA) on ROS generation. Mean fluorescence intensity is expressed as relative units with ± SD (n = 4). (**B**) MitoSOX-based flow cytometry detection of mitochondrial ROS in Jurkat cells following exposure to intestinal digests (0.32 μM OTA). Data in the histogram are presented as mean ± SD (n = 4). Significant differences between intestinal digests (VM, OTA, and OTA-VM) and the control, or between OTA and OTA-VM, are indicated as *p* < 0.001 (***). C: wheat flour bread; VM: bread with wheat flour and *Vaccinium myrtillus* L. 2%; OTA: bread with wheat flour contaminated with Ochratoxin A; OTA-VM: bread with wheat flour contaminated with Ochratoxin A and *Vaccinium myrtillus* L. 2%; TBHP: tert-Butyl hydroperoxide.

The histogram for the ROS level, as determined by flow cytometry, is shown in Figure 6 (A). According to the graph, cells exposed to intestinal OTA digest had a 4.1-relative-unit higher level of intracellular ROS than cells not exposed. Cells exposed to the OTA-VM digest exhibited a 0.3-relative-unit increase in ROS levels compared to non-exposed cells, along with a significant 75% reduction compared to OTA digest cells. In comparison to non-exposed cells, those exposed to the VM intestinal digest (1.38 ± 0.04 relative units) showed a minor significant increase, while C intestinal digest cells (1.01 ± 0.02 relative units) displayed no significant changes (Figure S3).

MitoSOX dye was utilized to assess how intestinal digests affected the production of ROS in the mitochondria. As shown in Figure 6B, in the case of all exposures, a significant increase in mitochondrial ROS was observed. The production of ROS increased the most with OTA digest, rising by 76% ± 2.87%. However, the addition of VM resulted in a significant decrease in the formation of ROS in the mitochondria (21% ± 0.67%) compared to OTA exposure, demonstrating its potent antioxidant impact also at the mitochondrial level (Figure S4).

### 3.8. Mitochondrial Mass Analysis

**Figure 7:**
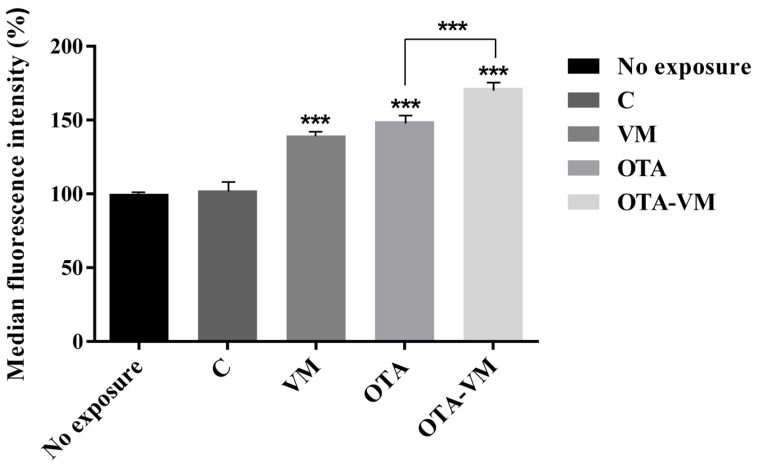
Effect of intestinal digest (0.32 μM OTA) on mitochondrial mass using Jurkat cells after 7 days of exposure. Median fluorescence intensity (MFI) (n = 4) of MitoTracker dye measured after incubation of Jurkat cells with intestinal digests. Significant differences between intestinal digests (VM, OTA, and OTA-VM) and control or OTA and OTA-VM are indicated as *p* < 0.001 (***). C: wheat flour bread; VM: bread with wheat flour and *Vaccinium myrtillus* L. 2%; OTA: bread with wheat flour contaminated with Ochratoxin A; OTA-VM: bread with wheat flour contaminated with Ochratoxin A and *Vaccinium myrtillus* L. 2%.

Mitochondrial mass was analyzed by incubating Jurkat-T cells exposed to intestinal digests with MitoTracker dye. After cells were exposed to OTA digest, a significant augmentation of 49 ± 1.45% in mitochondrial mass was discovered. Interestingly, after cells were exposed to OTA-VM, there was a significant 72 ± 1.76% increase in mitochondrial mass (Figure 7 and Figure S5).

## 4. Discussion

Over the past few years, a particular focus has been set on finding solutions to mitigate the adverse effects of mycotoxins. Previous studies have demonstrated the correlation between mycotoxins and the mitigation of their toxic effects using functional ingredients such as fermented milk whey 1%, pumpkin 1% [15], garlic 2% [27], and grape pomace 2% [26]. However, until now, bilberries, rich in polyphenols, have not been studied as a functional food to mitigate mycotoxin effects, more specifically OTA. The health benefits of polyphenols are influenced not only by their food sources but also by their stability, which can be affected by the matrix in which they are incorporated, post-harvest processing methods, and endogenous factors such as digestive enzymes and microbiota [28]. Numerous studies have shown the high stability of polyphenolic compounds after in vitro digestion [15,29].

The bilberry matrix incorporated as a functional ingredient in bread showed important antioxidant activity, correlated to other results from previous research in the same field. For the bilberry matrix used as a functional ingredient, the TPC was 62.29 ± 2.32 mg/g DW, which means 834.23 ± 31.11 mg/100 g FW. The results obtained for our samples were higher compared with those for bilberries from Montenegro [30], where the TPC ranged from 392 to 520 mg GAE/100 g FW, but was similar to the TPC found in bilberries from Serbia (890 ± 9 mg/100 g FW) [31,32]. The same trend was observed in the case of hydroxycinnamic acids, hydroxybenzoic acids, flavanols, and flavonols using LC–MS analysis. The concentration of gallic acid, a polyphenol with notable therapeutic properties such as anti-inflammatory, antioxidant, anti-angiogenic, and anti-cancer effects [33], was found to be higher in the studied sample (4.585 ± 0.275 mg/100 g DW) compared to a bilberry sample from Romania (3.419 ± 0.010 mg/100 g DW) [34].

High levels of anthocyanins were quantified in the examined sample, supporting their role in reducing inflammation, preventing cancer, and providing antimicrobial effects [35]. More specifically, in the case of Delphinidin 3-galactoside, a value of 214.96 ± 23.64 mg/100 g DW was obtained compared to another sample from Romania, for which 113.67 ± 11.00 mg/100 g DW was quantified. The same trend was observed in the case of other anthocyanins, demonstrating high concentrations for many of them: a value of 336.87 ± 23.47 mg/100 g DW was obtained for Delphinidin 3-glucoside compared to 119.86 ± 14.00 mg/100 g DW, 252.59 ± 17.68 mg/100 g DW was obtained for Cyanidin-3-O-galactoside compared to 91.85 ± 10.00 mg/100 g DW, 184.13 ± 11.05 mg/100 g DW was obtained for Petunidin 3-glucoside compared to 146.27 ± 18.00 mg/100 g DW, and 182.30 ± 12.76 mg/100 g DW was obtained for Malvidin 3-glucoside compared to 17.51 ± 3.20 mg/100 g DW [12].

After establishing the antioxidant properties of the functional ingredient, the evaluation of its possible beneficial role in reducing OTA bioaccessibility was performed. It was noted that the concentrations in the gastric compartment were significantly lower compared to those in the intestinal stage (Figure 2). This suggests that OTA released from bread predominantly occurs during the intestinal digestion phase, with pH playing a crucial role. Numerous studies investigating OTA absorption by in vitro gastrointestinal digestions showed differences of up to 90% for the gastric digest [15,36,37]. OTA bioaccessibility was examined with and without functional ingredients, showing a gastric bioaccessibility of 1% for the OTA digest and 0.4% for the OTA-VM digest and an intestinal bioaccessibility of 93% for the OTA digest and 78% for the OTA-VM digest, showing a significant beneficial VM effect on OTA-bioaccessibility. This reduction might be due to the increase of fiber and polyphenols by the addition of the VM, which might interact with OTA.

The mechanism by which OTA induces toxicity in vitro may involve multiple effects on various sub-cellular structures. Many studies have investigated the nephrotoxicity of OTA, but fewer have investigated the effects of OTA at the intestinal level, with in vitro studies showing induction of cell apoptosis and decreased cell viability [17]. The loss of cell viability upon OTA exposure may be attributed to compromised cell membrane integrity caused by ROS production [38]. To replicate the gastrointestinal in vitro barrier and provide a more accurate scenario, Caco-2 cells were differentiated concerning cell viability. Different dilutions of OTA digest resulted in an average reduction of 19.2% in cell viability (Figure 3). These results are supported by other investigations that demonstrate the concentration- and time-dependent reduction in cell viability of undifferentiated and differentiated Caco-2 cells exposed to varying concentrations of OTA (5 to 100 µM) [39,40]. This study also assessed bilberries’ cytoprotective in vitro function. Similar studies were performed on other cell lines (differentiated SH-SY5Y cells) exposed to intestinal digests containing low doses of OTA and functional ingredients such as pumpkin and fermented whey [41]. The incorporation of bilberries as a functional ingredient in contaminated bread resulted in a significant increase in cell viability—approximately 10% for each exposure time—compared to OTA digests, highlighting its potential to mitigate OTA-induced cytotoxicity.

The protective effect of certain compounds such as alpha-tocopherol has been demonstrated in Kidney Cell Line HK-2, OTA being known for its nephrotoxic effects [42]. Additionally, various natural compounds have been employed to reduce OTA toxicity, including resveratrol (RSV), luteolin (LUT), L-arginine (L-Arg), zinc, taurine (TAU), silymarin (Sil), selenomethionine (SeMet), melatonin, N-acetylcysteine (NAC), N-acetyl-tryptophan (NAT), gluconolactone (GA), glycyrrhizin (CAG), and astragalus polysaccharide (APS) [43]. In the present study, the protective effect of bilberry was observed, with a lower reduction of cell viability in the case of the digest containing both bilberry and mycotoxin compared to the digest containing mycotoxin alone (Figure 3).

Human lymphoblastic Jurkat cells were used to create a realistic scenario since they are a reliable immunological model for simulating human conditions in vitro [44]. The use of a 1:10 dilution of the intestinal digests exposed to this cell line supports the concept of a realistic scenario, as it reflects the assumption that approximately one-tenth of the ingested OTA concentration enters systemic circulation, a hypothesis supported by Frangiamone et al. [45]. Several mycotoxins have been revealed to be immunotoxic agents in vitro and in vivo [41]. It has been published that OTA had an inhibitory effect on the cell cycle in the G_1_, S, and G_2_/M phases [46], and it was found to induce G_0_/G_1_ phase cell cycle arrest in human renal proximal tubular HKC cells [47]. In this work, it was found that exposure to OTA can promote a possible arrest of the cell cycle at the G phase. By adding bilberries, there was a normalization of the condition, with statistical significance compared to the OTA condition, but without significance compared to the control.

Apoptosis is regulated through both extrinsic and intrinsic signaling pathways. Elevated levels of ROS can activate these cell death pathways, leading to both apoptosis and necrosis [48]. A similar trend was observed when evaluating the relationship between ROS and apoptosis following cell exposure to the mycotoxin. Specifically, a significant increase in cell death (10.79 ± 0.58%) was correlated with a substantial rise in ROS relative units, with digested OTA showing an increase of 4.1 relative units compared to the control.

Regarding the possibility of OTA causing oxidative stress, García-Pérez et al. [49] exposed HepG2 cells to concentrations ranging from 1 nM to 100 µM for 4 and 24 h, and showed an increase in ROS production between 27 and 35%. The secondary metabolites named anthocyanins, which provide fruit and vegetables their pigmentation, have recently attracted a lot of attention since they are potent antioxidants that can prevent ROS-induced oxidative damage [50]. The same trend was observed in the present work, noting that the addition of bilberries significantly decreased ROS production.

Mitochondria are crucial organelles responsible for maintaining normal metabolic cellular pathways, but they are susceptible to oxidative damage. Wang et al. [51] assessed IPEC-J2 cell ROS production exposed to OTA-increasing concentrations, observing a gradual increase of dichlorofluorescein fluorescence in these cells, suggesting excessive ROS was accumulated. Likewise, the results of the study showed that OTA exposure increased mitochondrial ROS production (35% more) when compared to the control digest. A decrease in mitochondrial ROS production in the OTA-VM digest (21% less) compared to the OTA digest was observed, confirming the antioxidant effect of bilberries against OTA also at the mitochondrial level (Figure 7).

Interestingly, when cells were exposed to VM digest, a significant increase in mitochondrial mass was observed. This result was also confirmed in the cancer cell line B16-F10, where the effect of increasing mitochondrial mass was observed upon exposure to an extract concentrated in anthocyanins [52]. The increased mitochondrial mass is a secondary response to respiratory chain deficiencies and may help improve energy homeostasis in the affected tissue [53].

## 5. Conclusions

This study focused on evaluating OTA bioaccessibility and cytotoxicity in vitro in bread, with or without the enrichment of lyophilized bilberries (2%). The decrease in OTA bioaccessibility and the increase of cell barrier viability in vitro upon the addition of bilberries were confirmed. At the intestinal level, the antioxidant effect of simulated bilberry ingestion on the impact of OTA on oxidative stress was evidenced. Improvement of apoptosis/necrosis, ROS, and mitochondrial ROS in immune cell homeostasis was demonstrated, thus confirming the use of bilberries as a potential bioactive ingredient. This research contributes significantly to the ongoing efforts to guarantee the safety of cereal products by highlighting the potential of using berries in food formulations to reduce mycotoxin toxicity.

## Figures and Tables

**Table 1:** Validation results of the LC-FLD method for the analysis of OTA.

Matrices	Linearity Range		r^2^	Matrix Calibration Curve	LOD/LOQ	
Barley Flour	0.05–5	(µg/g)	0.999	y = 224.14x + 2.1586	0.1/0.3	(ng/g)
Bread	0.3–5		1	y =219.18x + 4.0963	0.1/0.3	
Gastric digest	7.8–500	(µg/L)	0.991	y = 385.29x + 21.437	0.2/0.6	(µg/L)
Intestinal digest	156–2500		0.997	y = 419.11x + 19.094	0.2/0.6	

LOD, limit of detection. LOQ, limit of quantification. r^2^, regression coefficient.

**Table 2:** Recipes of the sample breads.

Ingredient	C (g)	VM (g)	OTA (g)	OTA-VM (g)
Wheat flour	59.7	57.7	52.7	50.7
Contaminated barley flour	-	-	7	7
Water	33	33	33	33
Salt	1.3	1.3	1.3	1.3
Sugar	2	2	2	2
Fresh yeast	4	4	4	4
Lyophilized bilberry	-	2	-	2
Total Quantity	100	100	100	100

C: wheat flour bread; OTA: bread with wheat flour contaminated with Ochratoxin A; OTA-VM: bread with wheat flour contaminated with Ochratoxin A and *Vaccinium myrtillus* L. 2%; VM: bread with wheat flour and *Vaccinium myrtillus* L. 2%.

**Table 3:** Determination of TPC and total antioxidant activity through DPPH assay for bilberry as dry weight.

Compound		Concentration
TPC	mg/g	62.29 ± 2.32
DPPH		114.48 ± 2.99

TPC—total polyphenolic content, expressed as mg gallic acid equivalent (GAE)/g dry weight (DW); DPPH—antioxidant activity through DPPH assay, expressed as mg Trolox equivalent (TE)/g DW (mean value ± SD, n = 3).

**Table 4:** Identification and quantification of bioactive compounds from bilberry as dry weight by LC/MS.

Category	Compound		Concentration
Hydroxycinnamic Acids	Chlorogenic acid	(µg/g)	1350.33 ± 27
Hydroxybenzoic Acids	Gallic acid	(µg/g)	45.85 ± 2.75
	Protocatechuic acid		25.18 ± 1.00
Flavanols	(+)-Epicatechin	(µg/g)	15.67 ± 0.78
	(−)-Catechin		0.22 ± 0.01
	Procyanidin A1		-
	Procyanidin B1		5.85 ± 0.06
	Procyanidin B2		46.56 ± 6.98
	Procyanidin B3		1.79 ± 0.05
	Procyanidin B4		9.73 ± 1.46
	Procyanidin C1		41.29 ± 1.24
	Procyanidin C2		-
Flavonols	Hyperoside	(µg/g)	210.31 ± 27.34
	Isoquercitrin		66.30 ± 0.66
	Quercitrin		24.84 ± 1.49
	Quercetol		5.10 ± 0.25
Anthocyanins	Delphinidin 3-galactoside	(mg/g)	2.15 ± 0.24
	Delphinidin 3-glucoside		3.37 ± 0.23
	Cyanidin-3-O-galactoside		2.53 ± 0.18
	Cyanidin 3-glucoside		-
	Cyanidin 3-arabinoside		1.37 ± 0.05
	Petunidin 3-glucoside		1.84 ± 0.11
	Malvidin 3-glucoside		1.82 ± 0.13

- Not found. Data are expressed as mean ± SD (n = 3).

**Table 5:** Quantification of Ochratoxin A in specific matrices using LC-FLD.

Matrices		OTA Concentration
Contaminated Barley Flour	(mg/kg)	149.05 ± 7.89
OTA-Bread		15.89 ± 0.13
OTA-VM-Bread		16.79 ± 0.55
OTA Gastric Digest	(µg/L)	15.22 ± 4.93
OTA-VM Gastric Digest		7.13 ± 3.18
OTA Intestinal Digest		1500.50 ± 1.22
OTA-VM Intestinal Digest		1287.58 ± 28.25

OTA: bread with wheat flour and barley flour contaminated with Ochratoxin A; OTA-VM: bread with wheat flour and barley flour contaminated with Ochratoxin A and lyophilized *Vaccinium myrtillus* L. 2%.

## Data Availability

Data are contained in the article.

## Supplementary Materials

The following supporting information can be downloaded at https://www.mdpi.com/article/10.3390/nu16183137/s1, Figure S1: Cell viability in differentiated Caco-2 cells following exposure to various dilutions of intestinal digests (3.2 μM OTA for no dilution) for 24 h (A), 48 h (B), 72 h (C), 92 h (D), 120 h (E) exposure times individually. Data graph lines and bars are the mean ± SD (n = 4). Significant differences between OTA intestinal digest exposure and control or OTA-VM intestinal digest exposure and control are indicated as *p* < 0.05 (*); *p* < 0.01 (**); *p* < 0.001 (***). Figure S2: Impact of intestinal digests (0.32 μM OTA) on apoptosis and necrosis pathways. Plots show results from the cell apoptosis assay following exposure of Jurkat cells to intestinal digests: control (A), OTA (B), OTA-VM (C), VM (D). Figure S3: Impact of intestinal digests (0.32 μM OTA) on ROS generation. Representative plots show cell count versus LOG fluorescence for 20,000 events analyzed using a flow cytometer to detect ROS level. Figure S4: MitoSOX-based flow cytometry detection of mitochondrial ROS in Jurkat cells after exposure to intestinal digests (0.32 μM OTA). The process for obtaining MitoSOX fluorescence intensity from Jurkat cells involves the following steps: (A) Selection of viable Jurkat cells based on side scatter (SSC) and forward scatter (FSC) characteristics, as shown in density plots. (B) Gating of singlet Jurkat cells based on forward scatter characteristics. (C) Measurement of median fluorescence intensity of Jurkat singlets using the B4 channel following exposures. Figure S5: Effect of intestinal digest (0.32 μM OTA) on mitochondrial mass. Steps to obtain median fluorescence intensity (MFI) from Jurkat cells/singlets are as follows: (A) Selection of live Jurkat cells based on side scatter (SSC) and forward scatter (FSC) characteristics. (B) Gating of singlet Jurkat cells using forward scatter characteristics, as shown in density plots. (C) Measurement of median fluorescence intensity of Jurkat singlets using the FITC channel.
