# Supplementary material for: Phytochemical Characterization of Bilberries and Their Potential as a Functional Ingredient to Mitigate Ochratoxin A Toxicity in Cereal-Based Products
Source: Nutrients. 2024 Sep 17;16(18):3137. doi: 10.3390/nu16183137 (PMC11435120; doi:10.3390/nu16183137)
Supplement: Supplementary file 1 [file nutrients-16-03137-s001.zip › nutrients-3155799-supplementary.pdf]

Supplementary figures

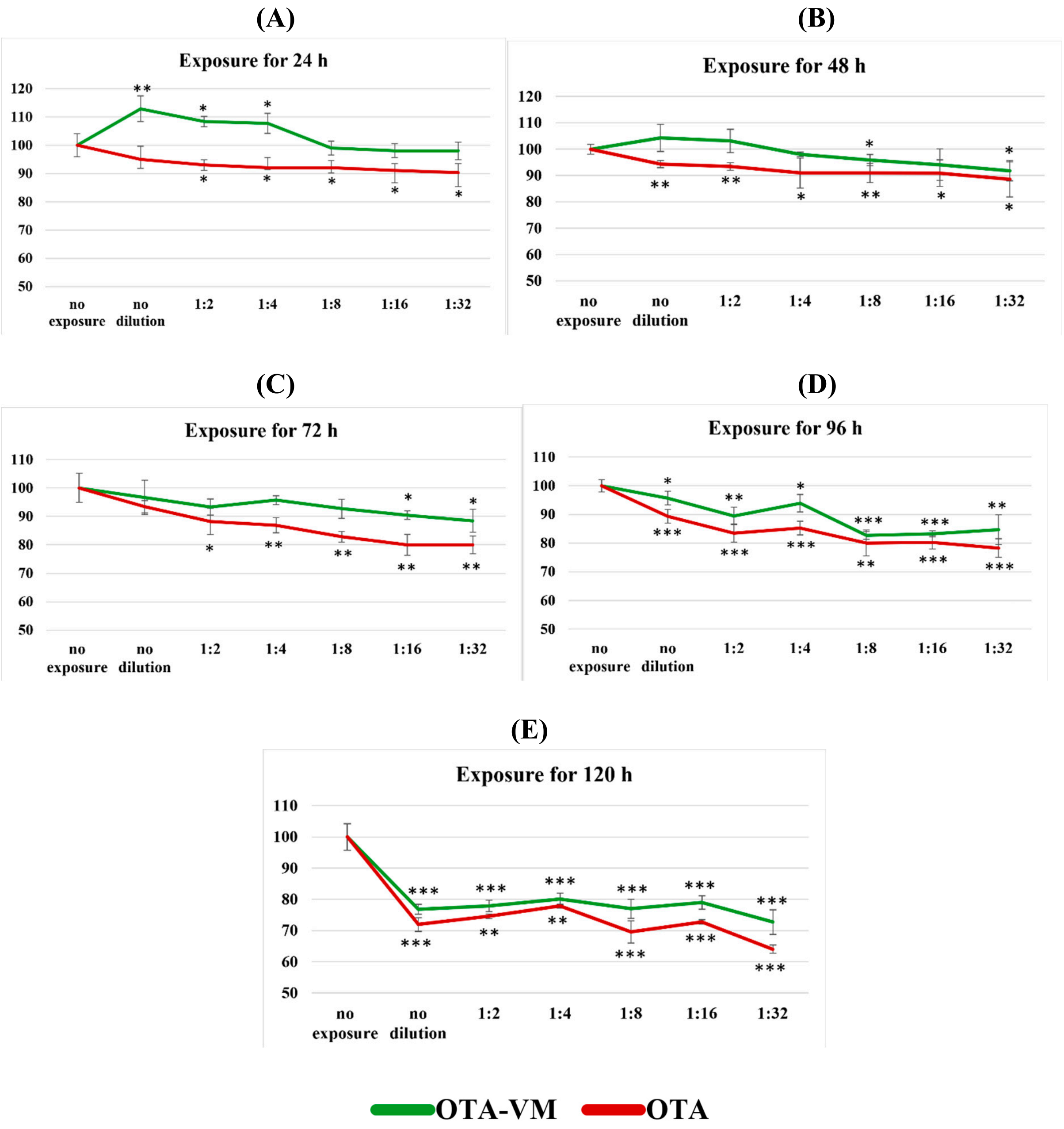

Figure S1. Cell viability in differentiated Caco-2 cells following exposure to various dilutions of intestinal di-gests (3.2 µM OTA for no dilution) for 24 h **(A)**, 48 h **(B)**, 72 h **(C)**, 92 h **(D)**, 120 h **(E)** exposure times individually. Data graph lines and bars are the mean ± SD (n = 4). Significant differences between OTA intestinal digest exposure and control or OTA-VM intestinal digest exposure and control are indicated as $p < 0.05$ (*); $p < 0.01$ (**); $p < 0.001$ (***).

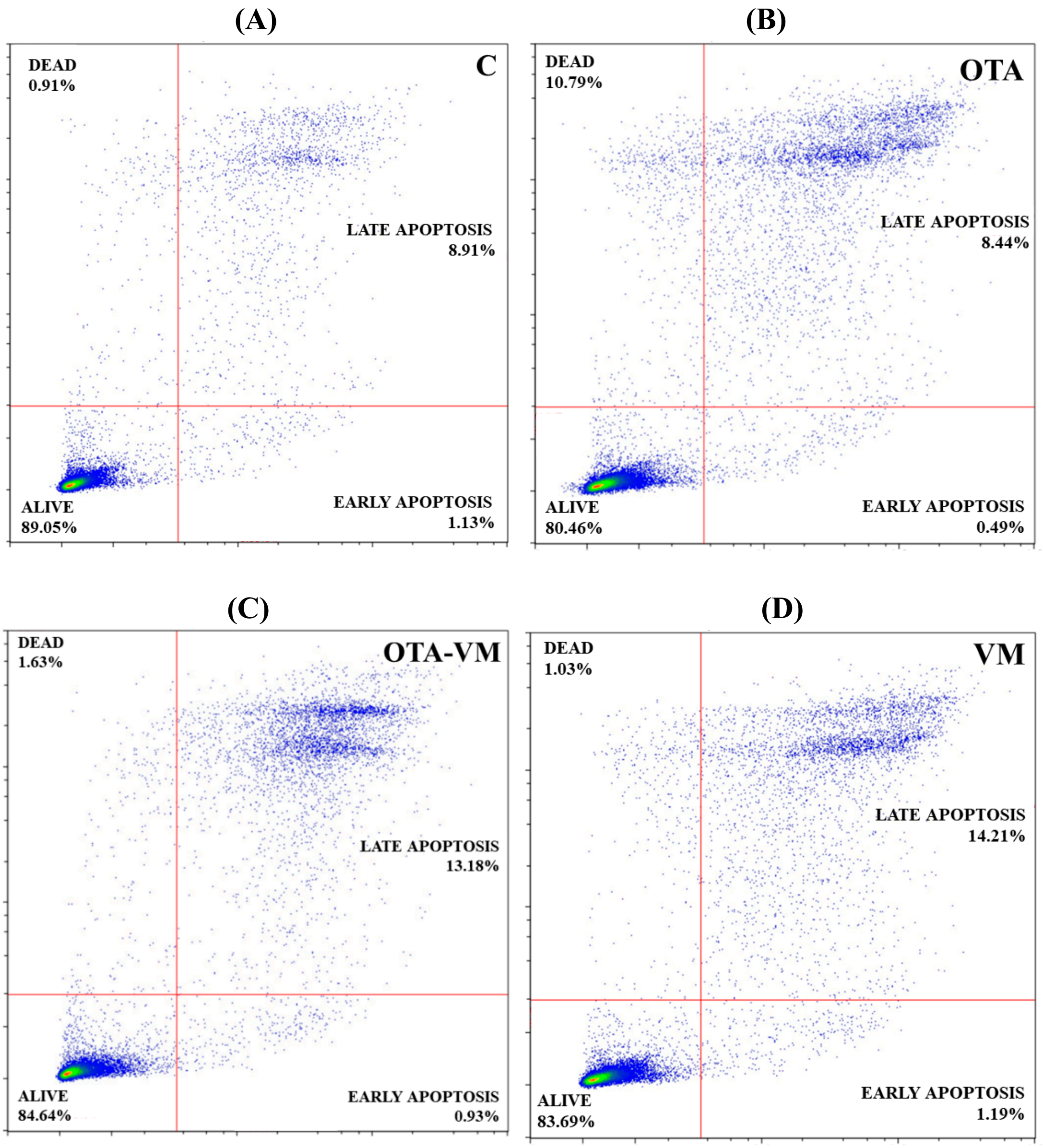

Figure S2. Impact of intestinal digests (0.32 μM OTA) on apoptosis and necrosis pathways. Plots show results from the cell apoptosis assay following exposure of Jurkat cells to intestinal digests: control **(A)**, OTA **(B)**, OTA-VM **(C)**, VM **(D)**.

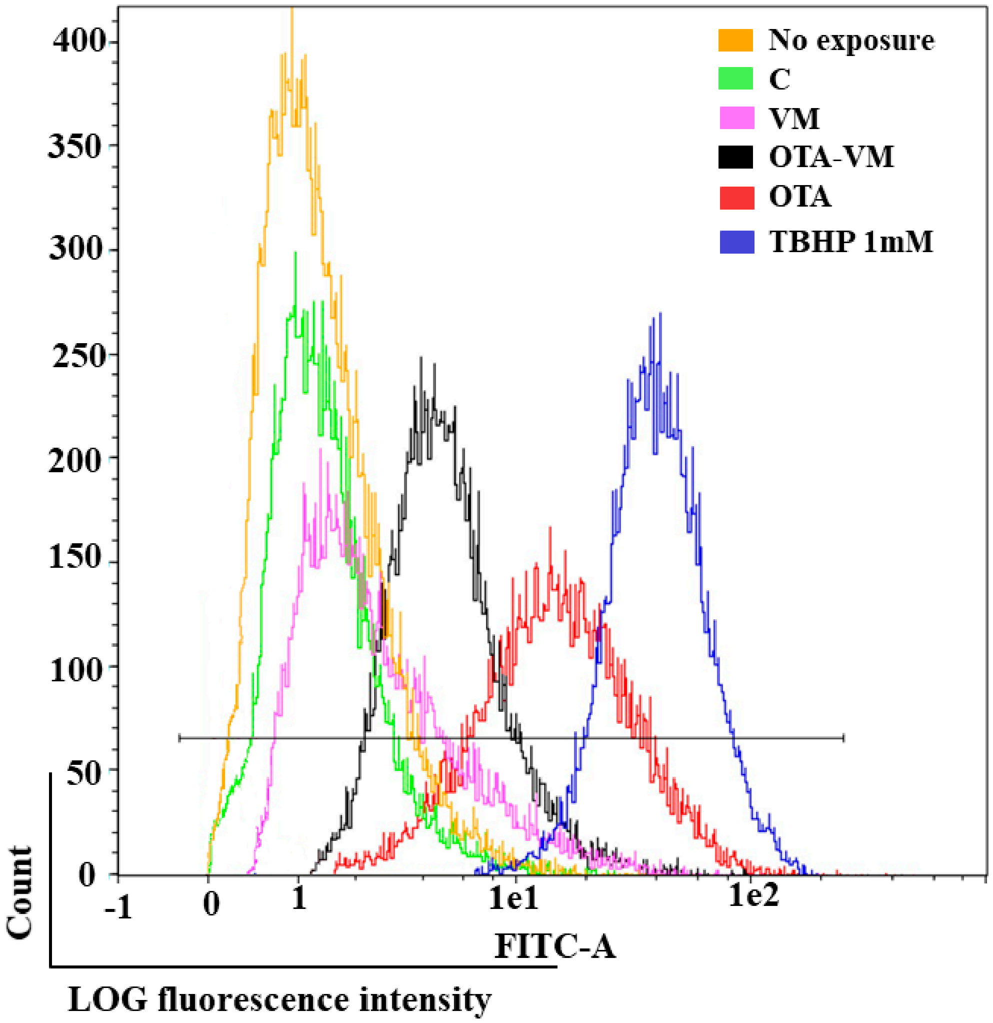

Figure S3. Impact of intestinal digests (0.32 µM OTA) on ROS generation. Representative plots show cell count versus LOG fluorescence for 20,000 events analyzed using a flow cytometer to detect ROS level.

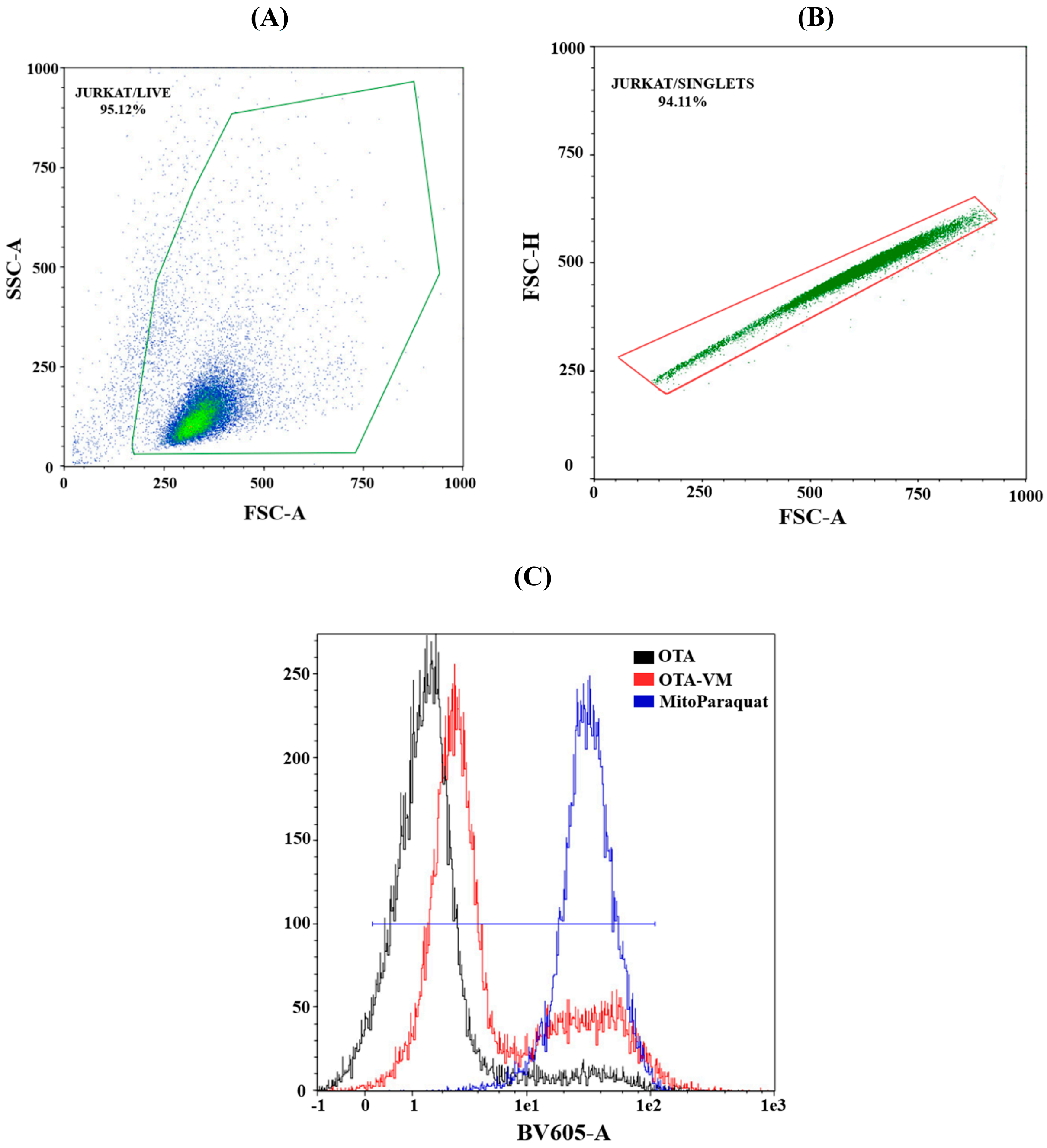

Figure S4. MitoSOX-based flow cytometry detection of mitochondrial ROS in Jurkat cells after exposure to intestinal digests (0.32 μM OTA). The process for obtaining MitoSOX fluorescence intensity from Jurkat cells involves the following steps: **(A)** Selection of viable Jurkat cells based on side scatter (SSC) and forward scatter (FSC) characteristics, as shown in density plots. **(B)** Gating of singlet Jurkat cells based on forward scatter characteristics. **(C)** Measurement of median fluorescence intensity of Jurkat singlets using the B4 channel following exposures.

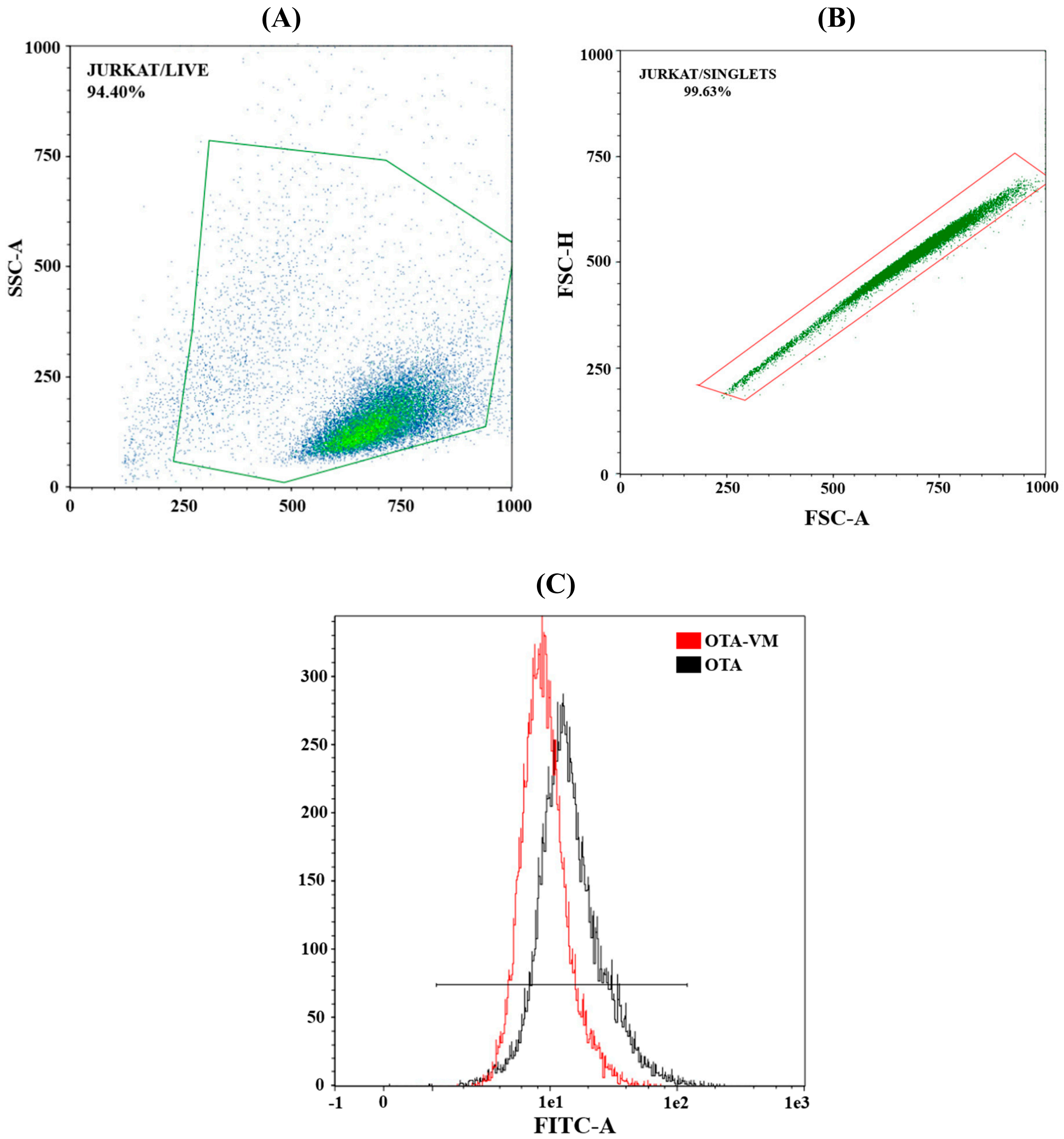

Figure S5. Effect of intestinal digest (0.32 µM OTA) on mitochondrial mass. Steps to obtain median fluorescence intensity (MFI) from Jurkat cells/singlets are as follows: **(A)** Selection of live Jurkat cells based on side scatter (SSC) and forward scatter (FSC) characteristics. **(B)** Gating of singlet Jurkat cells using forward scatter characteristics, as shown in density plots. **(C)** Measurement of median fluorescence intensity of Jurkat singlets using the FITC channel.
